# Mesenchymal Stem Cells and Co-stimulation Blockade Enhance Bone Marrow Engraftment and Induce Immunological Tolerance

**Published:** 2015-05-01

**Authors:** B. Rajeshkumar, P. Agrawal, M. Rashighi, R. F. Saidi

**Affiliations:** 1 *Department of Surgery, University of Massachusetts Medical School, Worcester, MA, USA*; 2 *Department of Medicine, University of Massachusetts Medical School, Worcester, MA, USA*; 3 *Division of Organ Transplantation, Department of Surgery, Alpert Medical School of Brown University, Providence, RI, USA*

**Keywords:** Organ transplantation, Immunosuppression, Infection

## Abstract

**Background:**

Organ transplantation currently requires long-term immunosuppression. This is associated with multiple complications including infection, malignancy and other toxicities. Immunologic tolerance is considered the optimal solution to these limitations.

**Objective:**

To develop a simple and non-toxic regimen to induce mixed chimerism and tolerance using mesenchymal stem cell (MSC) in a murine model.

**Methods:**

Wild type C57BL6 (H2D^k^) and Bal/C (H2D^d^) mice were used as donors and recipients, respectively. We studied to achieve tolerance to skin grafts (SG) through mixed chimerism (MC) by simultaneous skin graft and non-myeloablative donor bone marrow transplantation (DBMT) +/– MSC. All recipients received rapamycin and CTLA-4 Ig without radiation.

**Results:**

DBMT+MSC combined with co-stimulation blockage and rapamycin led to stable mixed chimerism, expansion of Tregs population and donor-specific skin graft tolerance. The flow cytometry analysis revealed that recipient mice developed 15%–85% chimerism. The skin allografts survived for a long time. Elimination of MSC failed to induce mixed chimerism and tolerance.

**Conclusion:**

Our results demonstrate that donor-specific immune tolerance can be effectively induced by non-myeloablative DBMT-MSC combination without any additional cytoreductive treatment. This approach provides a promising and non-toxic allograft tolerance strategy.

## INTRODUCTION

Successful solid organ transplantation currently requires chronic and long-term immunosuppression for essentially all patients. This is associated with multiple complications such as infection, malignancy and diabetes [1]. Immunologic tolerance is considered the optimal solution to these limitations. Mixed chimerism (MC) has been demonstrated to induce immune tolerance in rodent, non-human primates and human trials [2-9]. However, a disadvantage of the tolerance induction regimens is the requirement for a toxic conditioning, making it applicable only to recipients of living donor allografts. In addition, the regimen is not applicable to patients who are too sick to tolerate the pre-transplant conditioning regimen. Therefore, there is a need of nontoxic regimens to establish MC.

Mesenchymal stem cells (MSCs) have emerged as a promising cell population for immunomodulatory therapy in transplantation. These cells can easily obtain from bone marrow and other tissues such as umbilical cord blood, adipose tissue and muscle. MSCs have immunosuppressive properties and low immunogenicity [10, 11]. MSCs are capable of suppressing T effector cells and promote the development of Tregs.

The main objective of this study was to develop simple and non-toxic regimen to induce mixed chimerism and tolerance using MSC in a murine model.

## MATERIAL AND METHODS

Wild type C57BL6 (H2D^k^) and Bal/C (H2D^d^) mice were used as donors and recipients, respectively. All mice were 8–10 weeks old and housed under specific pathogen-free environment. The protocol was approved by animal care committee.

Isolation and Culture of MSC

Bone marrow-derived MSCs were isolated and cultured in our laboratory as described previously [11]. MSCs were characterized by their adherence, fibroblast-like morphology and capacity to differentiate into mesenchymal cell lineages—adipocytes, chondrocytes and osteoblasts.

Skin Graft

Full-thickness skin grafts were transplanted from donor to recipient mice at day 0. The skin graft of 1.0 × 1.5 cm was prepared and the subcutaneous and microvessels were carefully removed. A same size skin was removed from the recipients’ dorsum and the donor skin was fixed with glue and tape.

Flow Cytometry Analysis

Mulitocolor flow cytometry analysis was used to evaluate chimerism. In brief, peripheral blood was collected from the tail vein and nucleated cells were labeled with fluorochrome-tagged mAb after lysis of red blood cells.

Treatment Groups

**Figure 1:**
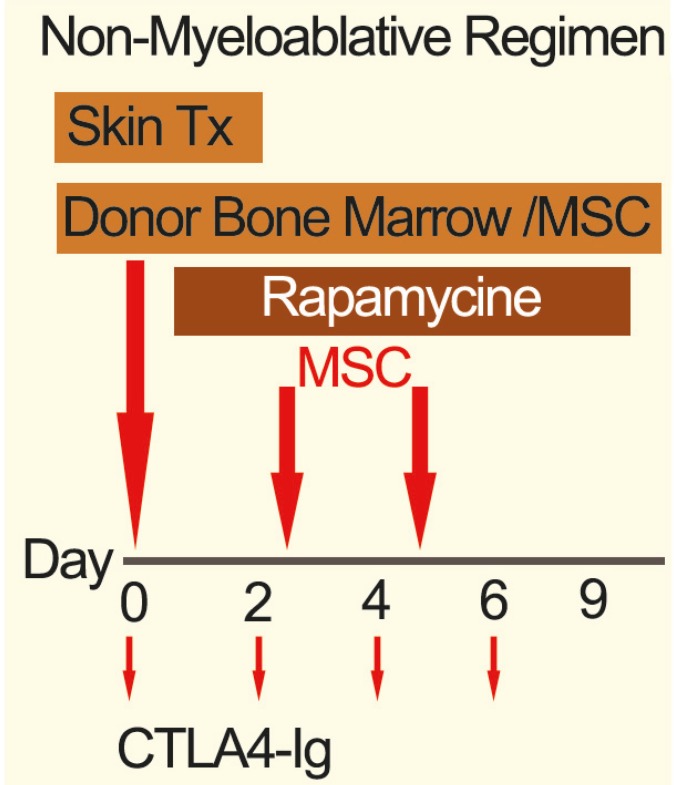
The non-myeloablivtive regimen which led to mixed chimerism and tolerance.

On the day of SG (day 0), bone marrow cells (250 × 10^6^ bone marrow cells per animal) and/or MSC (2 × 10^6 ^also repeated on days 2 and 4) from donors were prepared and infused via tail vein. After transplantation, recipients received rapamycin (2 mg/kg/day, ip) from day 0 to day 9, and CTLA-4 Ig (Bristol-Myers Squibb, NY; 250 microgram on day 0, 2, 4 and 6) (Fig 1). In the treatment control group, the recipient animals did not receive the full regimen. There were six animals in each group. Recipients were checked for mixed chimerism at 1, 2, 4, 6 and 8 weeks and then monthly after transplantation.

## RESULTS

**Figure 2:**
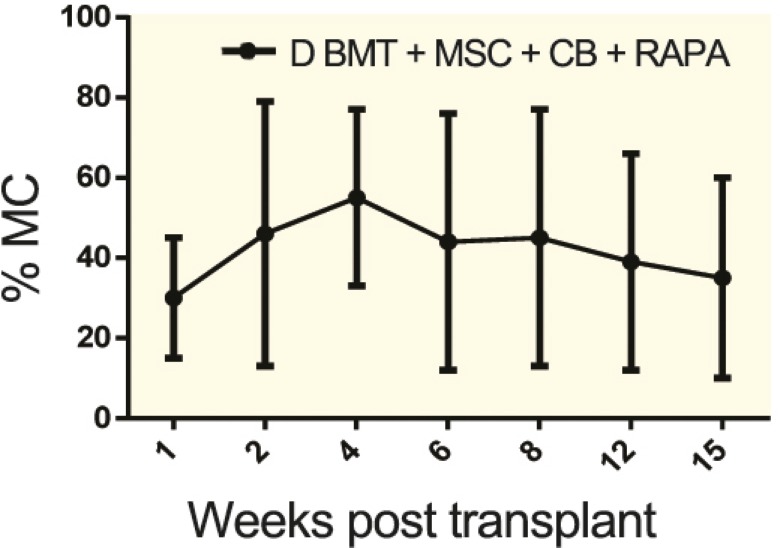
The percent of donor nucleated cells in peripheral blood of recipients (mean+-SD) was assessed by flow cytometry (MC; mixed chimerism, CB: costimulation blockade, rapa: rapamycin)

**Figure 3:**
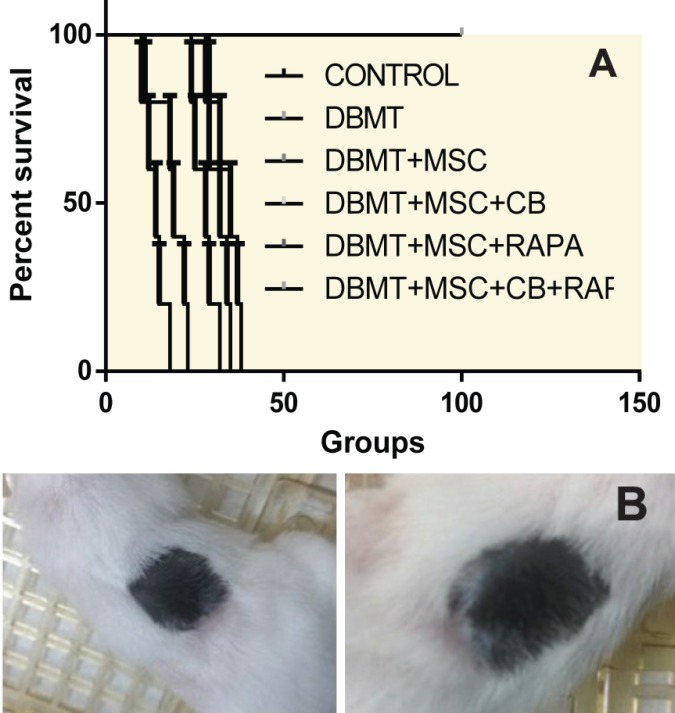
The survival of skin grafts in different groups (A), healthy skin graft and hair growth in the tolerant animals (B)

**Figure 4:**
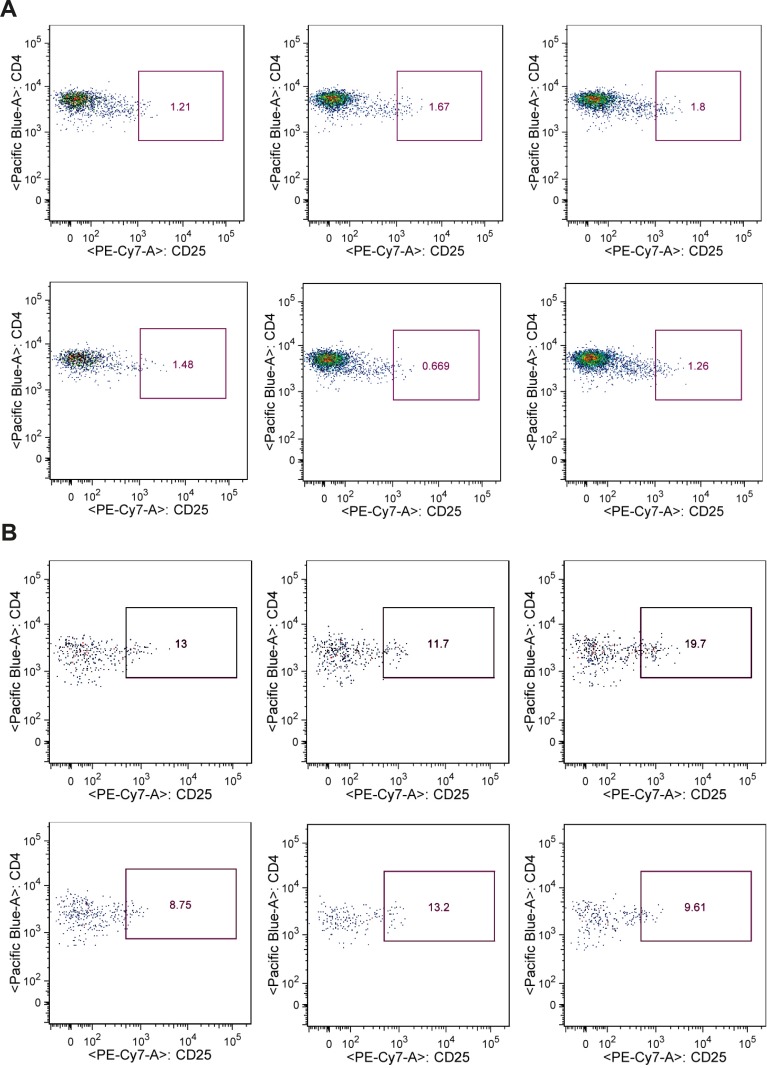
Compared to control (A ) Tregs population was expanded in tolerant group (B)

Only the group that received DBMT+MSC combination plus CTLA-4 Ig/rapamycin developed stable mixed chimerism (Fig 2) and donor-specific skin graft tolerance (Fig 3). The other groups, which did not receive the full regimen, did not become chimeric and although the skin graft survival was prolonged compared to the control group, they eventually rejected the skin graft. The flow cytometry analysis revealed that tolerant group mice developed 15%–85% chimerism levels among the multilineage. We also noted a significant expansion of Treg population in the tolerant mice (Fig 4). Recipient mice rejected third-party skin grafts. No case of graft versus host disease (GVHD) was observed in tolerant animals.

DBMT: donor bone marrow transplantation

MSC: mesenchymal stem cells

CB: co-stimulation blockade (CTLA-4 Ig)

Rapa: rapamycin

p<0.0001

DBMT: donor bone marrow transplantation

MSC: mesenchymal stem cells

CB: co-stimulation blockade (CTLA-4 Ig)

Rapa: rapamycin

## DISCUSSION

Solid organ transplantation has achieved great success during the past decade, largely because of advances in immunosuppression and control of acute rejection [1]. However, long-term graft survival has not changed in the past decade as the result of multiple factors including irreversible chronic rejection and patient death caused by the side effects of standard immunosuppressive drugs [1]. Tolerance induction thus remains a goal in the field of transplantation. Mixed chimerism through non-myeloablative DBMT has been shown to induce tolerance to kidney allografts in non-human primates and man. Based on rodent studies [2, 3], clinically relevant regimens were developed that permit the induction of mixed chimerism through non-myeloablative DBMT (to avoid GVHD) and renal allograft tolerance in large animals [4-6]. This approach was first applied to human recipients of living donor MHC-matched kidneys, whose renal disease resulted from multiple myeloma [7, 8]. More recently, the regimen was extended to HLA-mismatched kidney transplant recipients without myeloma [9]. A disadvantage of the current preparative regimen is the requirement for conditioning beginning six days prior to organ transplantation, making it applicable only to recipients of living donor allografts. In addition, the toxicity of regimen, especially radiation, makes it not applicable to other patients who are too sick to tolerate the pre-transplantation conditioning regimen.

We elected to use CTLA-4 Ig and rapamycin in our protocol as both agents are known to expand the Treg population [12, 13]. CTLA4-Ig is a fusion protein composed of the Fc region of the immunoglobulin IgG1 fused to the extracellular domain of CTLA-4. It is a molecule capable of binding to more avidity to CD80 (B7-1) than to CD86 (B7-2), which inhibits the co-stimulation of T cells. 

Co-stimulation blockade is an immunosuppression strategy that offers many benefits compared to conventional calcineurin inhibitor-based regimens. Chief among these advantages is a general lack of off-target toxicities such as nephrotoxicity, enabling better long-term graft function. More recently, the significant clinical potential of co-stimulation blockade was demonstrated in the phase-III BENEFIT trial of belatacept (a second-generation CD28 antagonist), which revealed that renal transplant patients treated with belatacept had superior long-term graft function compared to those treated with cyclosporine [14].

MSCs are a heterogeneous population of adult, fibroblast-like multipotent cells characterized by their ability to differentiate into tissues of mesodermal lineage including adipocytes, chondrocytes, and osteocytes [16-18]. Several *in vitro* and *in vivo* studies have documented the ability of MSC to polarize T cells toward regulatory phenotype [10, 11, 16]. MSCs have inhibitory effects on antigen-presenting cells, macrophages and B cell [16-18]. We also used bone marrow-derived MSCs due to its immunosuppressive and immunomodulative activities [16]. MSCs have immunoregulatory properties on both innate and adaptive immunity by which concurrent suppression of Th1, Th2 or T17 responses. There are several studies which reported the capability of MSCs to prolong allograft survival of different tissues such as skin, heart or liver [16-18]. There are few protocols of MSC-based therapy in solid-organ transplantation [18-20]. In this study, we developed a new strategy to induce donor-specific immune tolerance using MSC without additional toxic conditioning regimen. Our results showed that combination of bone marrow-derived MSCs and co-stimulation blockade in combination with donor BM induced long-term MC, Treg expansion and tolerance to allogeneic full-thickness skin graft. This regimen did not require radiation. Non-human primates studies need to confirm further applicability of this regimen to human.
